# The Clerical Resolution Online Widget (CROW): creating open-source software to make clerical review easier.

**DOI:** 10.23889/ijpds.v7i3.1790

**Published:** 2022-08-25

**Authors:** Hannah O'Dair, Anthony G Edwards, Craig Scott

**Affiliations:** 1 Office For National Statistics (UK)

## Objectives

Quality assessment is an integral part of linkage, often involving clerical (manual) review of records. Excel spreadsheets are commonly used to review record pairs, which can be difficult to read across the screen. We decided to create an adaptable piece of software that improved the user experience.

## Approach

Our approach was to utilise tkinter, a pythonic graphical user interface (GUI) package. Tkinter is easy to use and has simple syntax, making it a good choice for open-source code. The software takes in wide format datafiles and displays record pairs one at a time in an easy-to-compare format, allowing a user to select whether the items are a ‘match’ or a ‘non-match’. By adapting parameters in a configuration file, a user can very easily adapt the CROW to the variables available in each dataset. We have also developed capability for highlighting differences between record pairs.

## Results

Feedback from users indicates that as a result of using CROW, the clerical matching experience has improved vastly. Users reported that CROW increased accessibility, reducing eye strain and fatigue. CROW also increases efficiency, allowing projects to complete earlier than planned. On one key Labour Market Survey based linkage, the CROW led to an estimated 25% time saving. The ability to highlight the differences in records increases the accuracy and efficiency of decision making. We were successful in building adaptable software, that can be transferred to different datasets. It has been used in a wide range of projects across the ONS including key Covid-19 and Census 2021 linkage projects. The open-sourced nature of this software means it is freely available for use.

## Conclusion

In conclusion, CROW is an open-sourced piece of software for clerical review that is easy to use and adapt; it makes the experience of clerical review more pleasant as well as saving resources. It is a useful tool that enables quality assurance to be broadly implemented across the linkage domain.

